# 49. Impact of a Rapid Genotypic Platform for Gram-negative Bloodstream Infections, Paired with an Antimicrobial Stewardship Intervention, on Time to Optimal Antimicrobial Therapy

**DOI:** 10.1093/ofid/ofab466.251

**Published:** 2021-12-04

**Authors:** Madison Donnelly, Jennifer Walls, Katlyn Wood, Aiman Bandali

**Affiliations:** 1 Penn Medicine/Lancaster General Health, Lancaster, Pennsylvania; 2 Atlantic Health System, Summit, New Jersey

## Abstract

**Background:**

Gram-negative bacteremia is associated with significant morbidity and mortality. Development of an algorithm for antimicrobial selection, using institution-specific antibiogram data and rapid diagnostics (RDT), achieves timely and appropriate antimicrobial therapy. The objective of this study is to assess the impact of a pharmacy-driven antimicrobial stewardship initiative in conjunction with ePlex® BCID on time to optimal antimicrobial therapy for patients with gram-negative bloodstream infections.

**Methods:**

This retrospective, observational, single-center study included adult patients with a documented gram-negative bloodstream infection in whom the ePlex® BCID was employed. A pharmacist-driven antimicrobial stewardship intervention was initiated on December 1, 2020; pre-intervention (December 2019 – March 2020) was compared to the post-intervention (December 2020 – February 2020) period. The following organisms were included: *Citrobacter spp.*, *Escherichia coli, Klebsiella aerogenes/pneumoniae/oxytoca, Proteus spp, Enterobacter spp., Pseudomonas aeruginosa,* and *Acinetobacter baumannii*. Polymicrobial bloodstream infections or those who had an ePlex® panel performed prior to admission were excluded. The following clinical outcomes were assessed: time to optimal antimicrobial therapy, length of stay (LOS), and inpatient-30-day mortality.

**Results:**

One hundred and sixty-three met criteria for inclusion; 98 patients in the pre-intervention group and 65 patients in the post-intervention group. The mean Pitt Bacteremia Score was 1 in both groups (p=0.741). The most common organism identified by ePlex® BCID was *E. coli* (65.3% vs 70.8%; p=0.676). Eight *E. Coli* isolates were CTX-M positive; no other gene targets were detected. The most common suspected source of bacteremia was genitourinary (72.5% vs 72.3%; p=1.0). Time to optimal therapy was reduced by 29 hours [37 (31 – 55) vs. 8 (4 – 28); p=0.048). Length of stay and mortality was similar between groups.

**Conclusion:**

Implementation of a rapid blood culture identification panel along with an antimicrobial stewardship intervention significantly reduced time to optimal therapy. Further studies are warranted to confirm these results.

**Disclosures:**

**All Authors**: No reported disclosures

